# Analysis of CALR-mutated essential thrombocythemia as a distinct disease entity compared with JAK2 V617F-mutated and triple-negative patients

**DOI:** 10.55730/1300-0144.6184

**Published:** 2026-02-22

**Authors:** Gökhan Sami AYDIN, Elif AKSOY, İpek YÖNAL HİNDİLERDEN, Aynur DAĞLAR ADAY, Emine GÜLTÜRK, Meliha NALÇACI, Fehmi HİNDİLERDEN

**Affiliations:** 1 Division of Hematology, Department of Internal Medicine, Bakırköy Dr. Sadi Konuk Training and Research Hospital, İstanbul, Turkiye; 2 Division of Hematology, Department of Internal Medicine, Faculty of Medicine, İstanbul University, İstanbul, Turkiye; 3 Division of Medical Genetics, Department of Internal Medicine, Faculty of Medicine, İstanbul University, İstanbul, Turkiye

**Keywords:** CALR mutation, essential thrombocythemia, myeloproliferative neoplasm

## Abstract

**Background/aim:**

Calreticulin (CALR) mutations in essential thrombocythemia (ET) are associated with younger age, higher platelet counts, and lower thrombosis rates. The present study analyzes the demographic, laboratory, and clinical features of the CALR mutation and its prognostic impact.

**Materials and methods:**

The clinical impact of CALR mutations was assessed in 391 ET patients.

**Results:**

CALR-mutation patients were more commonly male than JAK2 V617F-positive and triple-negative patients. Age at diagnosis was similar across all groups, although patients with type 2 CALR mutations were younger than those with nontype 1/nontype 2 mutations. Compared with JAK2 V617F-positive patients, CALR-mutation patients had lower leukocyte counts (9.6 × 10^9^/L vs. 10.9 × 10^9^/L), lower hemoglobin (Hb) and hematocrit (Hct) levels, higher platelet counts (1078.5 × 10^9^/L vs. 858.1 × 10^9^/L), and lower total thrombosis rates (20.8% vs. 37.8%), while the CALR-mutated and triple-negative patients had lower venous thrombosis rates than in the JAK2 V617F-positive patients. The arterial thrombosis rate before and at the time of diagnosis was lower in the CALR-mutation patients than in the JAK2 V617F-positive patients, and the total venous thrombosis rate in patients aged <60 years at the time of diagnosis was lower in the CALR-mutation and triple-negative patients than in the JAK2 V617F-positive patients. Multivariable analysis revealed cardiovascular (CV) risk to be the only independent predictor of total thrombosis. Female gender, absence of CV risk, and platelet count ≥1000 × 10^9^/L were associated with a lower incidence of arterial thrombosis. Age <60 years was associated with lower risks of arterial and venous thrombosis. Overall, thrombosis-free, and leukemia-free survival were similar across all groups, while myelofibrosis-free survival was longer in the type 2 CALR-mutation group.

**Conclusion:**

The CALR mutation was lower among females, and was associated with lower leukocyte counts, and Hb and Hct levels, and with higher platelet counts. In multivariable analysis, the apparent protective association of CALR with thrombosis was not independent.

## 1. Introduction

Essential thrombocythemia (ET) is a myeloproliferative neoplasm (MPN) characterized by sustained thrombocytosis and a tendency for thromboembolic events or bleeding [1]. The calreticulin (CALR) gene was first described in 2013 as a somatic mutation in Janus kinase 2 (JAK2)- and myeloproliferative leukemia virus oncogene (MPL)-negative MPN [2]. CALR mutations account for 20–30% of all cases of ET. Compared to JAK2 V617F-positive ET, CALR-mutated ET is typically diagnosed at a younger age, with higher platelet counts, but lower hemoglobin (Hb) levels and leukocyte counts, and a lower risk of thrombotic complications [3]. Despite the relatively lower risk of thrombosis, CALR-mutated ET has been associated with a significantly higher risk of progression to myelofibrosis in most studies, while inconsistent results have been reported in others [4,5]. The present study investigates the clinical and laboratory correlates of 391 Turkish patients diagnosed with ET, and examines the impact of CALR gene mutations on clinical features and disease outcomes.

## 2. Materials and methods

### 2.1. Study population

Included in this retrospective descriptive study were 391 patients over 18 years of age who had been diagnosed with ET between 1995 and 2022 based on 2016 World Health Organization (WHO) criteria, and who were being followed up by the hematology departments of Bakırköy Dr. Sadi Konuk Training and Research Hospital, University of Health Sciences, (n = 222) and the İstanbul University Faculty of Medicine (n = 169). Patients with missing clinical or laboratory data or those lacking molecular analyses for CALR, JAK2 V617F, and MPL mutations were excluded from the study. MPL-positive patients were also excluded due to the small number of cases. Demographic, laboratory and clinical data at diagnosis, cardiovascular (CV) risk factors (smoking, hypertension, hyperlipidemia, diabetes, and family history of CV disease), mutational status (JAK2 V617F, MPL, CALR), clinical complications (thrombosis, bleeding, myelofibrotic transformation, leukemic transformation), mortality, overall survival (OS), thrombosis-free survival (TFS), leukemia-free survival (LFS), and myelofibrosis-free survival (MFS) data were collected from patient reports and electronic medical records. The medical histories of the participants were reviewed for thrombosis development before and after diagnosis and thrombotic events were classified as arterial or venous. Triple-negative ET patients were defined as those without the JAK2, MPL, or CALR driver mutations. The revised IPSET score based on age (≤60 years and >60), the presence of the JAK2 V617F mutation, and history of thrombosis were calculated for each patient. The study protocol was granted approval by the Local Ethics Committee of Bakırköy Dr. Sadi Konuk Training and Research Hospital, University of Health Sciences (August 1, 2022, 2022-15-07).

### 2.2. Genotyping for CALR exon 9 mutations

The Sanger sequencing technique (n = 222) or allele-specific Reverse transcription polymerase chain reaction (RT-PCR) (n = 169) were used to screen for CALR exon 9 mutations, including common type 1 (c.1092_1143del), type 2 (c.1154_1155insTTGTC), and other mutations.

### 2.3. Statistical analysis

The statistical analysis was conducted using IBM SPSS Statistics, version 26.0 (IBM Corp., Armonk, NY, USA). A one-way analysis of variance (ANOVA) was used to compare more than two groups of continuous data and post-hoc comparisons were made using Dunn’s test. The Mann–Whitney U test was used for the comparison of two independent groups of continuous data without normal distribution. Chi-square and Fisher’s exact tests were used for the comparison of categorical data. Multivariable Cox proportional hazards regression analysis was used for multivariate analysis. The Kaplan–Meier method was used for the analysis of OS, TFS, LFS, and MFS. For all tested hypotheses, two tailed p-values of <0.05 were considered statistically significant.

## 3. Results

### 3.1. Cohort

A total of 391 ET patients were included in the study, 63.4% (n = 248) of whom were women. The mean age at the time of ET diagnosis and the mean follow-up duration were 51.5 ± 16.5 years and 97.08 ± 65.7 months, respectively. At the time of diagnosis, the mean leukocyte count was 10.5 ± 4.1 (× 10^9^/L), the mean Hb and hematocrit (Hct) levels were 13.4 ± 1.6 g/dL and 40.8 ± 5.1%, respectively, and the mean platelet count was 904.7 ± 357.6 (× 10^9^/L). JAK2 V617F and CALR mutations accounted for 71.1% and 18.4% of the total, respectively, while 10.5% of the cohort was diagnosed triple-negative ET. Of the 72 patients with the CALR mutation, 37 (51.4%), 24 (33.3%), and 11 (15.3%) tested positive for type 1 CALR, type 2 CALR, and other CALR mutations (nontype 1 and nontype 2), respectively. The clinical and laboratory findings of the sample are summarized in Table 1. Patients were stratified as very low risk (18.9%, n = 74), low risk (35.5%, n = 139), moderate risk (5.6%, n = 22), and high risk (39.9%, n = 156) by the revised IPSET score. Of the total, 128 patients (32.7%) experienced a total of 136 thrombotic events, of which 86 were arterial, 37 were venous, and 13 were both arterial and venous. Furthermore, 93 thrombotic events were diagnosed before or at the time of ET diagnosis and 43 after ET diagnosis.

### 3.2. Comparison of clinical and laboratory characteristics of ET patients stratified by mutational status (JAK2 V617F-positive, CALR-mutated, and triple-negative ET)

Age at diagnosis was similar for the mutation groups, while the mean follow-up duration was significantly longer in the JAK2 V617F-positive group than the triple-negative ET patient group (101.8 ± 66.3 and 67.7 ± 34.6 months, respectively; p = 0.008). There was a predominance of males in the CALR-mutated ET group when compared with the JAK2 V617F-positive and triple-negative ET groups (54.2%. 34.2%, and 22%, respectively; p = 0.001) (Table 1). The mean leukocyte count (× 10^9^/L) at diagnosis was lower in the CALR-mutated ET group than the JAK2 V617F-positive ET group (9.6 ± 3.1 and 10.9 ± 4.5, respectively; p = 0.029). There was no difference between the mean leukocyte counts of the other groups. Mean Hb (g/dL) and Hct (%) levels were significantly lower in the CALR-mutated and triple-negative ET groups than the JAK2 V617F-positive ET group (12.7 ± 1.8, 13.1 ± 1.6 and 13.7 ± 1.6 respectively; p < 0.001, and 38.1 ± 5.1, 39.7 ± 4.8 and 41.7 ± 4.9, respectively; p < 0.001). The mean platelet count (× 10^9^/L) at diagnosis was higher in the CALR-mutated ET group than the JAK2 V617F-positive group (1078.5 ± 468.3 and 858.1 ± 306.4 respectively; p < 0.001). No significant difference was noted between the platelet counts of the other groups. The mean LDH level (U/L) at diagnosis was significantly higher in the JAK2 V617F-positive and CALR-mutated ET groups than in the triple-negative ET group (314.6 ± 185.7, 321.5 ± 128.9 and 232.1 ± 61.2 respectively; p = 0.010).

Significantly larger spleens (mm) were observed in the JAK2 V617F-positive ET group than the triple-negative ET group (132.6 ± 29.8 and 121.8 ± 14.5, respectively; p = 0.016). No significant difference in spleen size was observed between the other groups.

Splenomegaly was significantly more frequent in the JAK2 V617F-positive group when compared with the triple-negative ET group (32.4% and 9.8%, respectively; p = 0.004).

The prevalence of hematologic and solid malignancies before or after ET diagnosis was greater in the JAK2 V617F-positive and CALR-mutated ET groups than in the triple-negative ET group (9.7%, 16.6%, and 0%, respectively; p = 0.010). No significant difference in bleeding incidence and transformation to myelofibrosis was noted between the JAK2 V617F-positive, CALR-mutated, and triple-negative ET groups. A greater proportion of patients in the JAK2 V617F-positive and CALR-mutated ET groups required cytoreductive therapy than in the triple-negative ET group (86.7%, 87.5%, and 70.7%, respectively; p = 0.023). The cytoreductive and antithrombotic drugs prescribed are listed in Table 1.

### 3.3. Comparison of CALR-mutated ET patients with type 1 and type 2, and nontype 1 and nontype 2

The mean age at diagnosis was significantly lower in the type 2 CALR-mutated ET patients than in the nontype 1 and nontype 2 CALR-mutated ET patients (44.2 ± 17.7 and 60.1 ± 9.5, respectively; p = 0.026) (Table 2).

The proportion of patients treated with interferon was significantly higher in the type 1 and type 2 CALR-mutated ET groups than in nontype 1 and nontype 2 CALR-mutated ET groups (35.1%; 37.5% and 0.0%, respectively; p = 0.043).

### 3.4. Thrombotic events

Of the ET patients who developed thrombotic events before or at the time of diagnosis, 14.7% (n = 58), 6.3% (n = 25), and 2.5% (n = 10) developed arterial thrombosis, venous thrombosis, and both arterial and venous thrombosis, respectively. Of the ET patients who developed thrombotic events after ET diagnosis, 7.1% (n = 28), 3% (n = 12), and 0.8% (n = 3) developed arterial thrombosis, venous thrombosis, and both arterial and venous thrombosis, respectively. The most common sites of arterial thrombosis among the patients diagnosed with thrombotic events before or at ET diagnosis were cerebral arteries (n = 26, 6.6%) and coronary arteries (n = 24, 6.1%). Among patients diagnosed with thrombotic events after ET diagnosis, the most common site of arterial thrombosis was the coronary (n = 16, 4.3%) and cerebral arteries (n = 10, 2.5%). The most common forms of venous thrombosis among patients diagnosed with thrombotic events before or at ET diagnosis were splanchnic vein thrombosis (SVT) (n = 16, 4.1%), deep vein thrombosis (DVT) (n = 10, 2.5%), and cerebral vein thrombosis (n = 6, 1.5%).

The sites of venous thrombosis among patients diagnosed with thrombotic events after ET diagnosis were SVT (n = 4, 1%) and DVT (n = 4, 1%). The sites of thrombosis are summarized in Table 3.

The incidence of total thrombosis was lower in the CALR-mutated ET group than in the JAK2 V617F-positive ET group (20.8% and 37.8%, respectively; p = 0.004). The incidence of venous thrombosis was lower in the CALR-mutated and triple-negative ET groups than in the JAK2 V617F-positive ET group (5.6%, 2.4%, and 15.5% respectively; p = 0.009). The incidence of arterial thrombosis before or at ET diagnosis was lower in the CALR-mutated ET group than in the JAK2 V617F-positive ET group (5.6% and 21.2%, respectively; p = 0.005). Venous thrombosis was more likely to develop before or at ET diagnosis in the JAK2 V617F-positive ET group than in the CALR-mutated and triple-negative ET groups (11.2%, 4.2%, and 2.4%, respectively; p = 0.055).

We also evaluated the impact of age on the incidence of thrombosis in the mutation groups. In CALR-mutated and triple-negative patients aged ≤60 years at diagnosis, the incidences of total thrombosis and venous thrombosis were lower than in JAK2 V617F-positive patients (16%, 12.5%, and 4.8%, respectively; p = 0.003; and 6%, 3.1%, and 20.8%, respectively; p = 0.004). There was no difference in the incidence of arterial thrombosis between the mutation groups (p > 0.05).

In ET patients aged ≤60 years at diagnosis, the incidence of total thrombosis and venous thrombosis before or at ET diagnosis was significantly lower in the CALR-mutated group than the JAK2 V617F-positive ET group (4% and 21.9%, respectively; p = 0.011; and 4% and 15.7%, respectively; p = 0.020).

Higher incidences of arterial thrombosis before or at ET diagnosis were observed in JAK2 V617F-positive patients than in CALR-mutated and triple-negative patients (12.9%, 2%, and 6.3%, respectively; p = 0.057).

For patients aged >60 years, the incidences of total, arterial, and venous thrombosis were similar between the JAK2 V617F-positive, CALR-mutated, and triple-negative ET groups.

### 3.5. Multivariate analysis

A Cox regression analysis was conducted to investigate the impact of female sex, age ≤60 years at diagnosis, platelet count ≥1000 (× 10^9^/L), leukocyte count <11 (× 10^9^/L), CV risk factors, and CALR and JAK2 V617F mutations on the incidence of total thrombosis, arterial thrombosis, and venous thrombosis developing before, at or after ET diagnosis.

#### 3.5.1. Multivariate analysis of ET patients developing thrombosis before or at diagnosis

In multivariate analysis, JAK2 V617F mutation lost its significance for total and arterial thrombosis (HR 0.59; 95% CI (0.22–1.56); p = 0.297 and HR 1.29; 95% CI (0.44–3.79); p = 0.641). In univariate analysis, JAK2 V617F-positive patients showed a tendency for the development of venous thrombosis, while in multivariate analysis, no independent effect on development of venous thrombosis was noted for the JAK2 V617F mutation (Table 4).

Age ≤60 years at diagnosis and platelet count ≥1000 (× 10^9^/L) had no significant impact on the incidence of total thrombosis, contrary to the protective effect demonstrated in univariate analysis (Table 4). In multivariate analysis, CV risk was identified as an independent risk factor and female sex as a protective factor for the development of total thrombosis (HR 3.24; 95% CI (1.64–6.39); p = 0.001 and HR 0.46; 95% CI (0.20–0.79); p = 0.004).

Female sex, absence of CV risk, and age ≤60 years at diagnosis remained independently associated with a lower incidence of arterial thrombosis in multivariate analysis (Table 4**)** (HR 0.42, 95% CI (0.23–0.75); p = 0.004). Platelet count ≥1000 (× 10^9^/L) was a significant protective factor for the development of arterial thrombosis (HR 0.46, 95% CI (0.22–0.97); p = 0.042).

Multivariate analysis revealed aged ≤60 years at diagnosis to be a risk factor for the development of venous thrombosis (HR 5.43, 95% CI (1.79–16.41); p = 0.003).

#### 3.5.2. Multivariate analysis of ET patients developing thrombosis after diagnosis

After adjusting for confounding variables, female sex remained independently protective against development of total and arterial thrombosis (HR:0.39, 95% CI (0.20–0.79); p = 0.006 and HR:0.27, 95% CI (0.11–0.60); p = 0.013, respectively) (Table 3).

In univariate and multivariate analysis, no parameter was noted to affect the risk of venous thrombosis.

### 3.6. Survival

**Figure 1:**
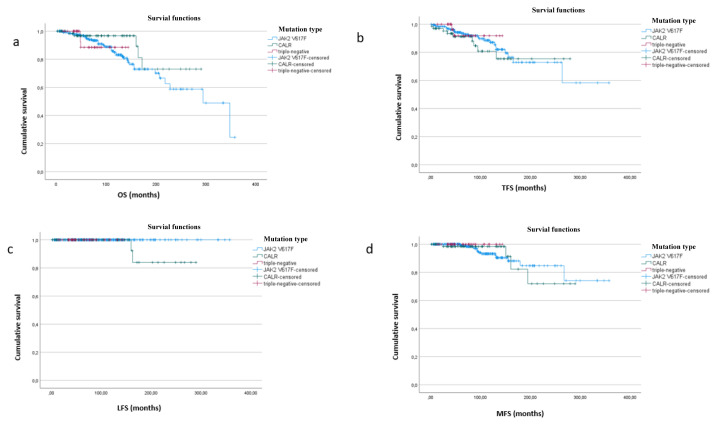
Survival outcomes in ET stratified by mutation status (n = 391). **a**. OS similar in patients with triple-negative, JAK2 V617F, and CALR mutations (132 months (95% CI: 121–144 months), 260 months (95% CI: 234–286 months), and 251 months (95% CI: 221–282 months), respectively; p = 0.486). **b**. TFS showing no difference between triple-negative, JAK2 V617F-positive, and CALR-mutated ET (135 months (95% CI: 125–146 months), 274 months (95% CI: 240–309 months), and 229 months (95% CI: 199–259 months), respectively; p = 0.860). **c**. The mean LFS was not reached in JAK2 V617F-positive and triple-negative ET patients, whereas 269 months (95% CI: 243–295 months) in for CALR-mutated ET. **d**. MFS was 313 months (95% CI: 287–338 months) in JAK2 V617F-positive and 254 months (95% CI: 222–286 months) in CALR-mutated ET patients, while MFS was not reached in triple-negative ET (p = 0.835).

The mean OS, TFS, and LFS of the total cohort were 266 months (95% CI: 242–289), 281 months (95% CI: 253–309), and 350 months (95% CI: 339–360), respectively. The mean OS and TFS of the JAK2 V617F-positive, CALR-mutated, and triple-negative ET groups were similar (p > 0.05). The mean LFS was not reached in the JAK2 V617F-positive and triple-negative ET groups (Figure 1).

The mean duration of MFS of the total cohort was 310 months (95% CI: 287–333), and did not differ between the mutation groups (p > 0.05) (Figure 1).

**Figure 2:**
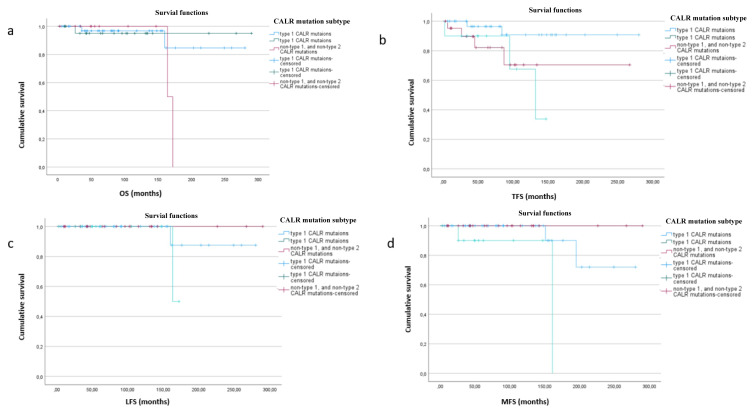
Survival data in ET stratified by CALR mutation subtype. **a**. OS did not differ in ET, stratified by the presence of type 1, type 2, and nontype 1, and nontype 2 CALR mutations (mean 257 months (95% CI: 227–287 months), 276 months (95% CI: 251–302 months), and 168 months (95% CI: 160–176 months), respectively; p = 0.277). **b**. TFS was similar between patients with type 1, type 2, nontype 1, and nontype 2 CALR mutations (mean 260 months (95% CI: 233–286 months), 203 months (95% CI: 215–270 months), and 116 months (95% CI: 86–146 months), respectively; p = 0.091). **c**. Comparison of LFS in patients with type 1 (mean 265 months, 95% CI: 237–292 months), type 2 (not reached), and nontype 1 and nontype 2 CALR mutations (mean 167 months, 95% CI: 161–173 months) (p = 0.398). d. MFS was inferior in patients with nontype 1 and nontype 2 CALR mutations (mean 147 months, 95% CI: 112–183 with respect to type 1 (mean 251 months 95% CI: 217–285) and type 2 mutations (not reached) (p = 0.006).

When stratified according to CALR mutation types, the mean OS, TFS, and LFS values did not differ (p > 0.05) (Figures 2a–2c), while the mean MFS of patients with the type 2 CALR mutation was significantly longer than for the type 1 and nontype 1 and nontype 2 mutations (p = 0.006) (Figure 2d).

## 4. Discussion

The present study compares the clinical findings, laboratory parameters, and patient outcomes of a large cohort of ET patients with long follow-up durations, stratified by driver mutation status (JAK2 V617F mutation, CALR mutation, or triple-negative). In comparison with previous studies in the literature, the frequency of CALR-mutated and triple-negative patients in our cohort was similar, while the frequency of the JAK2 V617F mutation was higher.

The mean age at diagnosis in the present study did not differ among ET patients when stratified by mutation status. Ojeda et al. reported their JAK2 V617F-positive ET patient group to be older than their triple-negative ET group, while the CALR-mutated ET patients showed no difference for age with respect to mutation status [6]. Previous studies have reported CALR-mutated ET patients to be younger than JAK2 V617F-positive and triple-negative ET patients [5,7,8].

There was a greater predominance of male patients in the CALR-mutated ET group when compared to the JAK2 V617F-positive and triple-negative ET groups. This finding is supported by some previous studies, while others report a similar sex distribution among the mutation groups [4–8].

In our cohort, CALR-mutated ET patients exhibited lower Hb and Hct levels and leukocyte counts but higher platelet counts, whereas triple-negative patients had lower Hb and Hct levels. Overall, our findings related to Hb and Hct levels are largely concordant with previous reports, while discrepancies exist with respect to leukocyte and platelet counts across the mutation subgroups [4,6,9]. Tefferi et al. and Ojeda et al. reported higher leukocyte counts in JAK2 V617F-positive patients when compared with CALR-mutated patients, while no such difference was observed by Elala et al. [4,6,9]. Furthermore, Ojeda et al. reported higher platelet counts in their CALR-mutated ET patients than in their JAK2 V617F-positive and triple-negative ET groups; however, the platelet counts were comparable between the CALR-mutated and triple-negative patients in the present study [6].

There have been few studies to date analyzing LDH levels with respect to ET mutational status [10,11]. Concurring with the findings of Rotunno et al., LDH levels in our cohort were higher in the CALR-mutated and JAK2 V617F-positive group than in the triple-negative group [10]. In another study, analyzing 212 ET patients, however, LDH levels did not differ between the mutation groups [11].

The association between mutation status and splenomegaly in ET has been previously investigated [7,10,11]. In a study of 576 ET patients conducted by Rotunno et al., splenomegaly was more common in CALR-mutated patients than in triple-negative patients, while no difference was observed between the CALR-mutated and JAK2 V617F-positive patients [10]. No splenomegaly comparison between JAK2 V617F-positive and triple-negative patients was conducted in that study [10]. In other studies, the frequency of splenomegaly did not differ according to mutational status (JAK2 V617F mutation, CALR mutation, MPL mutation, and triple-negative) [7,11]. In the present study, the frequencies of splenomegaly and increased spleen size were greater in the JAK2 V617F-positive ET patients than in the triple-negative patients, while no significant difference in spleen size was observed between the other groups.

In the present study the incidence of total thrombosis was lower in the CALR-mutated ET patients than JAK2 V617F-positive ET patients, in line with two previous studies [5,9]. In contrast to these two studies, in which a higher incidence of total thrombosis was observed in the JAK2 V617F-positive ET group compared to the triple-negative ET group, no such difference was noted in the present study.

There has been one previous report to date investigating the incidence of venous and arterial thrombosis in ET patients by mutation status [5]. The incidence of venous thrombosis was higher in the JAK2 V617F-positive group than in the CALR-mutated and triple-negative groups in the present study. Furthermore, the incidence of arterial thrombosis before or at diagnosis was higher in our JAK2 V617F-positive group than in our CALR-mutated group. In contrast to our study, Gangat et al. reported no difference in the incidence of arterial thrombosis before or at diagnosis, nor in venous thrombosis when ET patients were stratified by mutation status [5].

To the best of our knowledge, Gangat et al. performed the only study to date investigating the relationship between mutation status and thrombosis stratified by age <60 and ≥60 years at ET diagnosis [5]. In their study of 300 ET patients, the incidence of total and venous thrombosis before or at ET diagnosis was similar between CALR-mutated and JAK2 V617F-positive patients aged <60 years. In the present study, ET patients were stratified in terms of age ≤60 and >60 years at diagnosis based on revised IPSET [12], and in contrast to Gangat et al., the incidence of total and venous thrombosis at ET diagnosis was lower in the patients aged ≤60 years in the CALR-mutated and JAK2 V617F-positive groups in the present study. On the other hand, the two studies were consistent in reporting that the incidence of arterial thrombosis before or at ET diagnosis was similar for CALR-mutated and JAK2 V617F-positive patients [5].

In the present study, the incidence of total and arterial thrombosis was similar between the CALR-mutated and JAK2 V617F-positive ET groups, which differed from the reported lower incidence of total and arterial thrombosis among CALR+ ET patients (aged <60 years) than JAK2 V617F+ ET patients (aged <60 years) reported by Gangat et al. [5]. In accordance with the findings of Gangat et al., the incidence of venous thrombosis after ET diagnosis in patients aged ≤60 were similar for the CALR-mutated and JAK2 V617F-positive groups in the present study [5], and the incidences of total, arterial, and venous thrombosis were similar between the CALR-mutated and JAK2 V617F-positive ET patients aged >60 years [5].

The results of multivariate analyses in studies investigating the impact of clinical and laboratory characteristics and mutation status on thrombosis in ET are conflicting [5,13–17]. In our univariate analysis, the incidence of total thrombosis before or at diagnosis was significantly lower in the CALR-mutated group than the JAK2 V617F-positive group, although the protective significance of the CALR mutation disappeared in multivariate analysis.

Our multivariate analysis identified CV risk as an independent risk factor, while female sex had a protective effect against total thrombosis before or at diagnosis. In contrast, Bertozzi et al. reported no impact of CV risk and sex on total thrombosis [18]. Multivariate analyses revealed CV risk factors to be the strongest predictors of thrombosis, while female sex consistently showed a protective effect. Mutation status alone did not retain independent significance after adjusting for clinical variables, suggesting that thrombosis risk in ET is multifactorial and not solely mutation-driven.

In contrast with our study, in which the JAK2 V617F mutation lost its significance on arterial thrombosis before or at ET diagnosis, the multivariate analysis in the study by Gangat et al. confirmed the presence of JAK2 V617F mutation as an independent risk factor for arterial thrombosis before or at ET diagnosis [14]. In the present study, the female sex remained an independent protective factor against the development of arterial thrombosis before or at diagnosis. Multivariate analysis revealed platelet count ≥1000 (× 10^9^/L) to be a significant protective factor against arterial thrombosis before or at diagnosis. In the multivariate analysis by Gangat et al. studying the impact of age >60 years, mutations, and the presence of CV risk factors on arterial thrombosis before or at diagnosis, it was demonstrated that the presence of CV risk factors and age >60 years remained independently associated with a higher incidence of arterial thrombosis [14]. In one study including a large series of ET patients, the JAK2 V617F mutation was identified as an independent risk factor for venous thrombosis before or at ET diagnosis [14]. In the present study, venous thrombosis before or at diagnosis was increased only in patients aged >60 years.

In the univariate analysis by Finazzi et al., the incidence of total thrombosis after ET diagnosis was lower in their CALR-mutated group when compared with the JAK2 V617F-positive ET group; however, the CALR mutation lost its protective significance in multivariate analysis [16]. In our univariate and multivariate analyses, the CALR mutation had no impact on total thrombosis risk following ET diagnosis. Female sex was the sole independent protective factor for total and arterial thrombosis after ET diagnosis in our multivariate analysis. In one Turkish study, aged >60 years was identified as an independent factor affecting the cumulative thromboembolic incidence after ET diagnosis [17].

In the present study, the characteristics of ET patients by CALR mutation subtype (type 1, type 2, and nontype 1 and nontype 2) were compared. The ET patients with type 2 CALR mutations were younger than those with nontype 1 and nontype 2 mutations. Interferon treatment was more common among those with type 1 and type 2 CALR mutations than those with nontype 1 and nontype 2 mutations. In contrast with the aforementioned findings, the study by Narlı Özdemir et al. reported ET patients with nontype 1 and nontype 2 CALR mutations to be younger than those with type 1 and type 2 mutations, and a similar interferon treatment rate for the CALR mutation subtypes [17]. In the present study, the myelofibrotic transformation rate was higher in patients with nontype 1 and nontype 2 CALR mutations than in those with type 1 and type 2 mutations, although the difference was not statistically significant (18.2%, 5.4%, and 0).

In a multicenter study conducted in Türkiye by Narlı Özdemir et al., significantly higher rates of myelofibrotic transformation were observed in nontype 1 and nontype 2 CALR-mutated ET patients [17]. The same study reported similar rates among the CALR mutation subtypes as the present study in terms of sex, complete blood count, revised IPSET score, and splenomegaly, thrombosis, and leukemic transformation rates. In the study by Andrikovics et al. analyzing 289 ET patients, mean age did not differ among the different CALR mutation subtypes [19].

In line with previous findings, no significant difference in OS was observed in the present study with respect to the driver mutation status (JAK2 V617F mutation, CALR mutation, or triple-negative) [13,19–21]. Moreover, the type of CALR mutation had no impact on OS in the present study.

The literature contains several studies comparing TFS between JAK2 V617F-positive and CALR-mutated ET, while others compare TFS between JAK2 V617F-positive, CALR-mutated, and triple-negative ET groups [10,17,18,22]. Some studies report shorter TFS for JAK2 V617F-positive ET patients than those with CALR-mutated ET [17,22], while others report worse TFS for JAK2 V617F-positive ET than CALR-mutated and triple-negative ET [10,18]. In contrast with the aforementioned findings, no difference in TFS was observed in the present study with respect to the mutation status. In the present study, the type of CALR mutation had no impact on OS.

There have been limited studies to date assessing MFS according to mutation status in ET patients [4,17,23]. In line with the findings of Al Assaf [23], no significant difference in MFS was noted between the CALR-mutated and JAK2 V617F-positive ET patients in the study, while none of our triple-negative patients transformed to MF. Previous studies have reported similar MFS in CALR-mutated and JAK2 V617F-positive ET patients [4,17]. In a previous Turkish study, the rate of fibrotic transformation in ET was higher among those with nontype 1 and nontype 2 CALR mutations, whereas MFS was similar irrespective of the CALR mutation subtype [17]. In contrast, a higher yet nonsignificant rate of fibrotic transformation for nontype 1 and nontype 2 CALR mutations, and a significantly longer MFS for type 2 CALR-mutated ET patients than the other two subtypes of CALR mutations were observed in the present study.

There is little available data on LFS stratified by mutation status in ET patients [4,23,24]. In the study of 84 ET patients conducted by Kim et al., no significant difference was noted between CALR-mutated and JAK2 V617F-positive patients in terms of LFS [24]. In the large patient series analyzed by Elala et al., there was little difference in the LFS durations reported for JAK2 V617F-positive, CALR-mutated, MPL-positive and triple-negative ET patients [4]. The study by Al Assaf et al. analyzing 160 ET patients revealed similar LFS durations between CALR-mutated and JAK2 V617F-positive patients, and no leukemic transformations were observed in triple-negative patients [23]. In line with the aforementioned study, no differences in LFS durations were noted between CALR-mutated, JAK2 V617F-positive, and triple-negative ET patients in the present study. Moreover, LFS durations were comparable among all the CALR-mutation subtypes.

## 5. Conclusion

In summary, CALR-mutated ET is characterized by distinct clinical and biological features, including a higher proportion of males, lower leukocyte counts and Hb and Hct levels, and higher platelet counts, as well as a lower overall risk of total and venous thrombosis. Furthermore, the incidence of arterial thrombosis before or at ET diagnosis is reduced in CALR-mutated ET. For total thrombosis before or at ET diagnosis, the presence of CV risk and female sex were identified as an independent risk factor and as a protective factor, respectively. In addition, female sex, absence of CV risk, and platelet count ≥1000 (× 10^9^/L) were associated with a lower incidence of arterial thrombosis, whereas age ≤60 years at diagnosis was protective against both arterial and venous thrombosis before or at ET diagnosis. Female sex was identified as the sole variable associated with a lower incidence of total and arterial thrombosis developing after ET diagnosis. Our multivariate analysis suggests that arterial thrombosis observed in patients aged >60 years may, at least in part, reflect age-related atherosclerotic burden and the presence of conventional CV risk factors rather than disease-specific mechanisms alone. Type 2 CALR-mutated ET is associated with longer MFS. Collectively, these findings support the identification of CALR-mutated ET as a distinct disease entity with unique molecular and clinical characteristics.

## Figures and Tables

**Table 1:** Clinical and laboratory characteristics of patients with essential thrombocythemia stratified by mutation status.

Parameters	ET (n = 391)	JAK2V617F (n = 278)	CALR (n = 72)	Triple-negative (n = 42)	p

Sex					
Female, n (%)	248 (63.4)	183 (65.8)	33 (45.8)	32 (78)	**0.001**
Male, n (%)	143 (36.6)	95 (34.2)	39 (54.2)	9 (22)	

Age at ET diagnosis, mean (±SD)	51.5 ± 16.6	52.5 ± 16.6	49.9 ± 16.5	47.6 ± 16.1	0.142
<60, n (%)	260 (66.5)	178 (64)	50 (69.4)	32 (78)	0.174
≥60, n (%)	131 (33.5)	100 (36)	22 (30.6)	9 (22)	

WBC at ET diagnosis, mean (±SD)	10.5 ± 4.1	10.9 ± 4.5	9.6 ± 3.1	9.9 ± 2.9	**0.029**

HB at ET diagnosis, mean (±SD)	13.4 ± 1.7	13.7 ± 1.6	12.7 ± 1.8	13.1 ± 1.6	**<0.001**

HCT at ET diagnosis, mean (±SD)	40.8 ± 5.1	41.7 ± 4.9	38.1 ±5.1	39.7 ± 4.8	**<0.001**

PLT at ET diagnosis, mean (±SD)	904.7 ± 357.6	858.1 ± 306.4	1078.5 ± 468.3	922.9 ± 381.8	**<0.001**

LDH at ET diagnosis, mean (±SD)	307.5 ± 169.6	314.6 ± 185.7	321.5 ± 128.9	232.1 ± 61.2	**0.010**

Spleen size at ET diagnosis, mean (±SD)	130.1 ± 26.9	132.6 ± 29.8	125.7 ± 18.2	121.8 ± 14.5	**0.016**
Splenomegaly, n (%)	109 (27.8%)	90 (32.4%)	15 (20.8%)	4 (9.8%)	**0.004**

CV Risk n (%)	254 (65)	193 (69. 4)	41 (56.9)	20 (48.8)	**0.010**

Revised IPSET-t score					
Very low risk, n (%)	74 (18.9)	-	46 (63.9)	28 (68.3)	
Low risk, n (%)	139 (35.5)	136 (48.9)	2 (2.8)	1 (2.4)	0.968
Intermediate risk, n (%)	22 (5.6)	-	16 (22.2)	6 (14.6)	
High risk, n (%)	156 (39.8)	142 (51.1)	8 (11.1)	6 (14.6)	

Bleeding n (%)	81 (20.7)	57 (20.5)	17 (23.6)	7 (17.1)	0.702

Thrombosis, n (%)	128 (32.7)	105 (37.8)	15 (20.8)	8 (19.5)	**0.004**
Arterial, n (%)	96 (24.5)	77 (27.7)	12 (16.7)	7 (17.1)	0.077
Venous, n (%)	48 (12.8)	43 (15.5)	4 (5.6)	1 (2.4)	**0.009**

Before or at diagnosis					
Thrombosis, n (%)	93 (23.7)	81 (29.2)	6 (8.3)	6 (14.6)	**0.005**
Arterial, n (%)	68 (17.4)	59 (21.2)	4 (5.6)	5 (12.2)	**0.005**
Venous, n (%)	35 (8.9)	31 (11.2)	3 (4.2)	1 (2.4)	**0.055**

After diagnosis					
Thrombosis, n (%)	43 (10.9)	32 (11.5)	9 (12.5)	2 (4.9)	0.597
Arterial, n (%)	31 (7.9)	21 (7.6)	8 (11.1)	2 (4.9)	0.455
Venous, n (%)	15 (3.8)	14 (5.1)	1 (1.4)	-	0.229^.^

Cytoreductive therapy, n (%)	335 (90.7)	241 (86.7)	63 (87.5)	29 (70.7)	**0.023**
Hydroxyurea, n (%)	307 (78.5)	224 (80.6)	56 (77.8)	26 (63.4)	**0.04**
Anagrelide, n (%)	70(17.9)	42 (15.1)	18 (25)	9 (22)	0.109
IFN, n (%)	62(15.8)	35 (12.6)	22 (30.6)	4 (9.8)	**<0.001**

Leukemic transformation, n %	4 (1)	2 (0.7)	2 (2.8)	-	0.149

Secondary malignancy, n %	39 (9.9)	27 (9.7)	12 (16.6)	-	**0.010**

Myelofibrotic transformation, n (%)	19 (4.9)	15(5.3)	4 (5.5)	-	0.441

**Table 2:** Sites of arterial and venous thrombosis before or at and after diagnosis among essential thrombocythemia patients.

	Before or at diagnosis n (%)	After diagnosis n (%)
Arterial thrombosis	58 (14.7)	28 (7.1)
Venous thrombosis	25 (6.3)	12 (3)
Both arterial and venous thrombosis	10 (2.5)	3 (0.8)
Arterial thrombosis sites		
Coronary arteries	33	19
Cerebrary arteries	35 (6.6)	12 (2.5)
Peripheral arteries	4 (1.3)	1
Abdominal arterial	3 (0.8)	3 (0.3)
Venous thrombosis sites		
Splanhnic venous	16 (4.1)	5 (1.3)
Deep venous thrombosis or/and Pulmonary emboli	13 (3.3)	8 (2.2)
Cerebral venous	6 (1.5)	4 (1.1)

**Table 3:** Multivariate analysis of risk factors for thrombosis before, at, and after diagnosis in all patients with essential thrombocythemia.

Before or at diagnosis	All thrombosis		Arterial thrombosis		Venous thrombosis	
Parameters	HR (95% CI)	p	HR (95% CI)	p	HR (95% CI)	p
Female sex	**0.46 (0.20–0.79)**	**0.004**	**0.42 (0.23–0.75)**	**0.004**	1.77 (0.85–3.72)	0.127
Diagnosis age <60	0.64 (0.37–1.10)	0.112	**0.29 (0.16–0.55)**	**0.001**	**5.43 (1.79–16.41)**	**0.003**
Diagnostic platelet count <1000 (×109/L)	1.69 (00.89–3.21)	0.106	**0.46 (0.22–0.97)**	**0.042**	0.77 (0.29–2.06)	0.613
Diagnostic leukocyte count <11 ×10^9^/L	0.87 (0.50–1.48)	0.608	1.60 (0.87–2.94)	0.131	0.61 (0.26–1.40)	0.245
CV risk factor	**3.24 (1.64–6.39)**	**0.001**	**3.18 (1.33–7.60)**	**0.009**	1.77 (0.78–3.99)	0.169
CALR mutation	2.86 (0.79–10.39)	0.109	0.26 (0.05–1.19)	0.084	1.28 (0.12–13.43)	0.833
JAK2V617F mutation	0.59 (0.22–1.56)	0.297	1.29 (0.44–3.79)	0.641	4.21 (0.54–32.51)	0.168
After diagnosis	All thrombosis		Arterial thrombosis		Venous thrombosis	
In the diagnosis parameters	HR (95% CI)	p	HR (95% CI)	p	HR (95% CI)	p
Female sex	**0.39 (0.20–0.79)**	**0.006**	**0.27 (0.11–0.60)**	**0.013**	0.45 (0.14–1.48)	0.194
Diagnosis age <60	1.58 (0.75–3.32)	0.228	1.03 (0.44–2.37)	0.942	1.58 (0.41–6.11)	0.505
Diagnostic platelet count <1000 (×10^9^/L)	1.22 (0.56–2.71)	0.610	1.34 (0.52–3.44)	0.534	1.61 (0.32–8.04)	0.560
Diagnostic leukocyte count <11 ×10^9^/L	0.87 (0.43–1.74)	0.702	0.76 (0.31–1.77)	0.535	1.60 (0.40–6.34)	0.501
CV risk factor	0.54 (0.24–1.19)	0.128	0.52 (0.19–1.41)	0.201	1.64 (0.47–5.61)	0.431
CALR mutation	2.16 (0.49–9.60)	0.361	1.72 (0.33–9.05)	0.517	*	*

* not available.

**Table 4:** Clinical and laboratory characteristics of patients with essential thrombocythemia by CALR mutation subtype.

Parameters	CALR type 1 (37)	CALR type 2 (24)	CALR other type (11)	p

Sex				
Female, n (%)	16 (43.2)	11 (45.8)	6 (54.5)	0.804
Male, n (%)	21 (56.8)	13 (54.2)	5 (45.5)	

Age at ET diagnosis, mean (±SD)	50.6 ± 16	44.2 ± 17.7	60.1 ±9.5	**0.026**
<60, n (%)	26 (70.3)	19 (79.2)	5 (45.5)	0.131
≥60, n (%)	11 (29.7)	5 (20.8)	6 (54.5)	

WBC at ET diagnosis, mean (±SD) (×10^9^/L)	10.1 ± 3.2	9.5 ± 3	8.2 ± 2.9	0.145

HB at ET diagnosis, mean (±SD)	12.8 ± 1.6	12.3 ± 2.1	12.9 ± 2	0.527

HCT at ET diagnosis, mean (±SD)	38.8 ± 4.1	36.8 ± 5.9	38.7 ± 6.4	0.279

PLT at ET diagnosis, mean (±SD) (×10^9^/L)	1051.8 ± 307.8	1192.8 ± 568.7	918.7 ± 640.1	0.246

LDH at ET diagnosis, mean (±SD)	340.1± 128.9	318± 134.3	266.6 ± 109.4	0.251

Spleen size at ET diagnosis, mean (±SD)	127.8 ± 19.5	121 ± 14.7	128.7 ± 20	0.298

CV risk n (%)	19 (51.4)	14 (58.3)	8 (72.7)	0.447

Revised IPSET risk level				
Very low risk, n (%)	23 (62.2)	18 (75)	5 (45.5)	0.597
Low risk, n (%)	2 (5.4)	-	-	
Intermediate risk, n (%)	8 (21.6)	4 (16.7)	4 (36.4)	
High risk, n (%)	4 (10.8)	2 (8.3)	2 (18.2)	

Thrombosis, n (%)	5 (13.3)	6 (25)	4 (36.4)	0.216
Arterial, n (%)	3 (8.1)	2 (8.3)	1 (9.1)	0.2632
Venous, n (%)	2 (5.4)	4 (16.7)	3 (27.3)	0.360

Before or at diagnosis arterial, n (%)	3 (8.1)	-	1 (9.1)	0.732
Before or at diagnosis venous, n (%)	1 (2.7)	2 (8.3)	-	0.091

After diagnosis arterial, n (%)	2 (5.4)	3 (12.5)	3 (27.3)	0.486
After diagnosis venous, n (%)	-	1 (4.2)	-	

Myelofibrotic transformation, n (%)	2 (5.4)	-	2 (18.2)	0.092

Bleeding, n (%)	7 (18.9)	6 (25)	4 (36.4)	0.480

Cytoreductive treatment, n (%)	32 (86.5)	21 (87.5)	10 (90.9)	0.740

## References

[1] Grinfeld J Nangalia J Baxter EJ Wedge DC Angelopoulos N Classification and personalized prognosis in myeloproliferative neoplasms The New England Journal of Medicine 2018 379 15 1416 1430 10.1056/NEJMoa1716614 30304655 PMC7030948

[2] Klampfl T Gisslinger H Harutyunyan AS Nivarthi H Rumi E Somatic mutations of calreticulin in myeloproliferative neoplasms The New England Journal of Medicine 2013 369 25 2379 2390 10.1056/NEJMoa1311347 24325356

[3] Tefferi A Vannucchi AM Barbui T Essential thrombocythemia: 2024 update on diagnosis, risk stratification, and management American Journal of Hematology 2024 99 4 697 718 10.1002/ajh.27216 38269572

[4] Elala YC Lasho TL Gangat N Finke C Barraco D Calreticulin variant stratified driver mutational status and prognosis in essential thrombocythemia American Journal of Hematology 2016 91 5 503 506 10.1002/ajh.24338 26890983

[5] Gangat N Wassie EA Lasho TL Finke C Ketterling RP Mutations and thrombosis in essential thrombocythemia: prognostic interaction with age and thrombosis history European Journal of Haematology 2015 94 1 31 36 10.1111/ejh.12389 24889737

[6] Ojeda MJ Bragós IM Calvo KL Williams GM Carbonell MM *CALR*, *JAK2* and *MPL* mutation status in Argentinean patients with *BCR-ABL1*-negative myeloproliferative neoplasms Hematology 2018 23 4 208 211 10.1080/10245332.2017.1385891 28990497

[7] Tefferi A Wassie EA Lasho TL Finke C Belachew AA Calreticulin mutations and long-term survival in essential thrombocythemia Leukemia 2014 28 12 2300 2303 10.1038/leu.2014.148 24791854

[8] Sun C Zhou X Zou Z-J Guo H-F Li J-Y Clinical manifestation of calreticulin gene mutations in essential thrombocythemia without Janus Kinase 2 and MPL mutations: a Chinese cohort clinical study Chinese Medical Journal 2016 129 15 1778 1783 10.4103/0366-6999.186641 27453224 PMC4976563

[9] Tefferi A Wassie EA Guglielmelli P Gangat N Belachew AA Type 1 versus type 2 calreticulin mutations in essential thrombocythemia: a collaborative study of 1027 patients American Journal of Hematology 2014 89 8 E121 E124 10.1002/ajh.23743 24753125

[10] Rotunno G Mannarelli C Guglielmelli P Pacilli A Pancrazzi A Impact of calreticulin mutations on clinical and hematological phenotype and outcome in essential thrombocythemia Blood 2014 123 10 1552 1555 10.1182/blood-2013-11-538983 24371211

[11] Misawa K Yasuda H Araki M Ochiai T Morishita S Mutational subtypes of *JAK2* and *CALR* correlate with different clinical features in Japanese patients with myeloproliferative neoplasms International Journal of Hematology 2018 107 6 673 680 10.1007/s12185-018-2421-7 29464483

[12] Haider M Gangat N Lasho T Abou Hussein AK Elala YC Validation of the revised International Prognostic Score of Thrombosis for Essential Thrombocythemia (IPSET-thrombosis) in 585 Mayo Clinic patients American Journal of Hematology 91 4 390 394 10.1002/ajh.24293 26799697

[13] Chen C-C Gau J-P Chou H-J You J-Y Huang C-E Frequencies, clinical characteristics, and outcome of somatic *CALR* mutations in *JAK2*-unmutated essential thrombocythemia Annals of Hematology 2014 93 12 2029 2036 10.1007/s00277-014-2151-8 25015052

[14] Gangat N Karrar O Al-Kali A Begna KH Elliott MA One thousand patients with essential thrombocythemia: the Mayo Clinic experience Blood Cancer Journal 2024 14 1 10 10.1038/s41408-023-00972-x 38238303 PMC10796913

[15] Aleem A Algahtani A Alshaman L Aldawsari N Alqahtani F Clinical and pathologic characteristics of essential thrombocythemia (ET) patients harboring calreticulin (CALR) mutations Blood 2022 140 Supplement 1 12282 12283 10.1182/blood-2022-169941

[16] Finazzi G Carobbio A Guglielmelli P Cavalloni C Salmoiraghi S Calreticulin mutation does not modify the IPSET score for predicting the risk of thrombosis among 1150 patients with essential thrombocythemia Blood 2014 124 16 2611 2612 10.1182/blood-2014-08-596676 25323688

[17] Narlı Özdemir Z İpek Y Patır P Ermiş G Çiftçiler R Impact of *CALR* and *JAK2V*617F mutations on clinical course and disease outcomes in essential thrombocythemia: a multicenter retrospective study in Turkish patients Turkish Journal of Hematology 2024 41 1 26 36 10.4274/tjh.galenos.2024.2023.0430 38433449 PMC10918406

[18] Bertozzi I Peroni E Coltro G Bogoni G Cosi E Thrombotic risk correlates with mutational status in true essential thrombocythemia European Journal of Clinical Investigation 2016 46 8 683 689 10.1111/eci.12647 27271054

[19] Andrikovics H Krahling T Balassa K Halm G Bors A Distinct clinical characteristics of myeloproliferative neoplasms with calreticulin mutations Haematologica 2014 99 7 1184 1190 10.3324/haematol.2014.107482 24895336 PMC4077079

[20] Bai J Sheng M Xing W Bai J Li R *Calr vs. JAK2 vs. MPL* mutations in the progression of Philadelphia-negative myeloproliferative neoplasms Blood 2017 130 Supplement 1 5283

[21] Prejzner W Mital A Bieniaszewska M Leszczyńska A Szymańska A Clinical characteristics of essential thrombocythemia patients depend on the mutation status Acta Haematologica Polonica 2020 51 4 230 235

[22] Pérez Encinas MM Sobas M Gómez-Casares MT Abuin Blanco A Noya Pereira MS The risk of thrombosis in essential thrombocythemia is associated with the type of *CALR* mutation: a multicentre collaborative study European Journal of Haematology 2021 106 3 371 379 10.1111/ejh.13561 33275803

[23] Al Assaf C Van Obbergh F Billiet J Lierman E Devos T Analysis of phenotype and outcome in essential thrombocythemia with CALR or JAK2 mutations Haematologica 2015 100 7 893 897 10.3324/haematol.2014.118299 25934766 PMC4486223

[24] Kim BH Cho Y-U Bae M-H Jang S Seo E-J *JAK2* V617F, *MPL*, and *CALR* mutations in Korean patients with essential thrombocythemia and primary myelofibrosis Journal of Korean Medical Science 2015 30 7 882 888 10.3346/jkms.2015.30.7.882 26130950 PMC4479941

